# The Best Protocol to Treat Equine Skin Wounds by Second Intention Healing: A Scoping Review of the Literature

**DOI:** 10.3390/ani14101500

**Published:** 2024-05-18

**Authors:** Gesiane Ribeiro, Lúcia Carvalho, João Borges, José Prazeres

**Affiliations:** 1 Faculty of Veterinary Medicine, Lusófona University, Campo Grande 376, 1749-024 Lisbon, Portugal; p6589@ulusofona.pt (L.C.); vetequestre@gmail.com (J.B.); vetstoestevao@gmail.com (J.P.); 2 Veterinary and Animal Research Centre (CECAV), Faculty of Veterinary Medicine, Lusófona University—Lisbon University Centre, Campo Grande 376, 1749-024 Lisbon, Portugal; 3 MED—Mediterranean Institute for Agriculture, Environment and Development, Évora University, Pólo da Mitra Apartado 94, 7006-554 Évora, Portugal

**Keywords:** equine wound management, equine wound healing, horses, exuberant granulation tissue

## Abstract

**Simple Summary:**

As in humans, the treatment of skin wounds is a common clinical practice in veterinary medicine, especially in horses. This subject still requires great attention from researchers due to animal welfare concerns and economic losses resulting from the prolonged treatment of chronic wounds. This study has reviewed the scientific literature to identify which types of therapy have been described for equine wound healing. After selecting the publications using pre-established eligibility criteria, 81 manuscripts were included for data extraction on the characteristics of the articles, treatments, evaluation types, and results. Although the literature on equine wound management is very vast, there was observed a lack of evidence for the adoption of a treatment protocol, and many treatments with controversial results.

**Abstract:**

Equine skin wound treatment continues to be a challenge for veterinarians. Despite being a frequent practice, it remains difficult to choose an evidence-based treatment protocol. This study aimed to comprehensively explore the literature and provide a scoping review of therapeutic strategies for equine skin wounds and identify knowledge gaps and opportunities for future research. This review was conducted using specific criteria to select literature that described methods to manage second intention wound healing. After removing duplicates and screening papers for suitability, 81 manuscripts were included for data extraction. Of these, 59 articles were experimental studies, 10 were case reports, 9 were case series, and 3 were clinical studies. The most frequent wound location was the distal limbs. Macroscopic assessment was the main tool used to evaluate treatment effectiveness. All of the case reports, case series, and clinical studies reported positive outcomes with regard to the treatment used, while only 36% of the experimental studies found significant healing improvement in treated wounds compared to control groups. It was found that there are many treatments that have exhibited controversial results, and there exists a lack of evidence for the adoption of specific treatment protocols.

## 1. Introduction

Wound healing is a vital process for the health of animals, but can be associated with several complications [1]. In horses, cutaneous wounds occur commonly and often require expensive and prolonged treatment because many of them are not amenable to primary closure due to massive tissue loss, excessive skin tension, extreme contamination, or undue time elapsed since injury [2].

Second intention wound healing in horses is associated with species-specific problems such as exuberant granulation tissue (“proud flesh”) and subsequent retardation of epithelialization and contraction, especially when the wounds are located on the distal aspect of the limbs [3]. The pathophysiology of “proud flesh” in horses is not completely clear, but several contributing factors have been described, such as the anatomy and function of the distal limb, high motion areas, low oxygen tension, chronic contamination, growth factors concentration, collagen synthesis and degradation, and a prolonged low grade of inflammation [4,5,6].

Wounds in horses also have the tendency to become infected due to various environmental factors such as fecal contamination, dirt, and plant debris, as well as foreign bodies. Due to the risk of infection, the main purpose of wound management is to reduce the presence of bacteria on the healing tissue, and to achieve this, wound lavage, debridement, dressing, and bandaging are common methods employed in wound management [7]. According to Freeman et al. [8], treating skin wounds in horses can be challenging due to wide variations in the type, location, and severity of different wounds, and the lack of primary evidence on best management practices. 

A scoping review is a type of knowledge synthesis that uses a systematic and iterative approach to identify and synthesize an existing or emerging body of literature on a given topic [9]. It may be a step before undertaking research or conducting another type of review, such as a systematic review [10]. This study aimed to comprehensively explore the literature and provide a scoping review of current therapeutic strategies for equine skin wounds, as well as to discuss knowledge gaps and opportunities for future research.

## 2. Materials and Methods

This review was conducted following the framework proposed by Arksey and O’Malley [11], the preferred reporting of items for systematic reviews and meta-analyses extension for scoping reviews (PRISMA-ScR) [12], and the updated methodological guidance for the conduct of scoping reviews [13].

### 2.1. Identifying the Research Question

The proposed research question “What is the best protocol for treating skin wounds in horses?” addresses the need to assess the published literature in the area of equine wound management as a preparatory step towards a larger project being conducted by Lusófona University regarding wound healing in horses.

### 2.2. Identifying Relevant Studies

The search was performed individually by two researchers in September 2023, and the electronic databases used were Scopus, Pubmed, and Web of Science. The search strategy used was: ((equine wound) OR (horses wound) AND (treatment OR care OR healing OR management)). Search filters were used for the date (2000–2023), language (English, French, Spanish, and Portuguese), and document types. 

Due to the differences between the electronic database filters, the document types were selected as follows: Scopus, article, review, and conference paper; Pubmed, case report, classical article, clinical study, clinical trial, clinical trial veterinary, comparative study, controlled clinical trial, guideline, meta-analysis, practice guideline, preprint, randomized controlled trial, review, systematic review, and validation study; and Web of Science, article, review article, and proceeding paper. 

### 2.3. Study Selection

After searching the databases, articles were imported into Rayyan software (https://rayyan.qcri.org/welcome, accessed on 25 September 2023), according to Mak and Thomas’ [10] recommendations. Rayyan can help identify duplicates and perform title/abstract screening. The software also allows for blinding the results of team members’ reviews to each other.

Studies that described or compared the management of equine skin wounds that healed for second intention were included. Articles included could have addressed protocols for lavage, debridement, dressing, local or systemic treatments, as well as herbal medicine and gas therapy. The population included was only horses.

Articles regarding the treatment of wounds associated with fractures, burns, tumors, and any kind of comorbidities were excluded. Also, treatments with acupuncture, homeopathy, music therapy, aromatherapy, and reiki were excluded.

Articles underwent two levels of screening and one level of data extraction. Screening and data extraction were performed independently by three reviewers. At all stages of screening and data extraction, conflicts were resolved by consensus. 

Level 1 screening was performed on titles and abstracts. All studies that met the eligibility criteria were included. Level 2 screening was performed on full-text articles. At this level, only articles that presented a treatment protocol with immediate clinical applicability were included for data extraction. 

Review articles were excluded because the extraction of several relevant data was not applicable.

### 2.4. Charting the Data

The full-text studies were independently analyzed by three researchers and relevant data were extracted, including the following:

- Authors and date
- Type of study
- Manuscript language
- Study location
- Participant count and total number of wounds
- Wound location
- Treatment groups (type of intervention and comparator)
- Outcomes measured
- Outcomes (intervention effects)

The location of the study was considered to be the place of origin of the first author of the publications. The type of study design extracted was based on the design used in the study, not the design reported by authors, and the following categories were used: experimental (skin wounds were surgically induced and there was a control group), clinical study (real-occurring skin wounds and there was a control group), and case report/case series (real-occurring skin wounds and there was no control group).

### 2.5. Collating, Summarizing, and Reporting Data

Study characteristics, the types of interventions, and the outcomes assessed are summarized descriptively in the text and the tables. Figures are also used to convey this information, where appropriate. Chart headings, publication categorizations, and classifications were determined, and a consensus was reached after discussion with all researchers. 

## 3. Results

**Figure 1:**
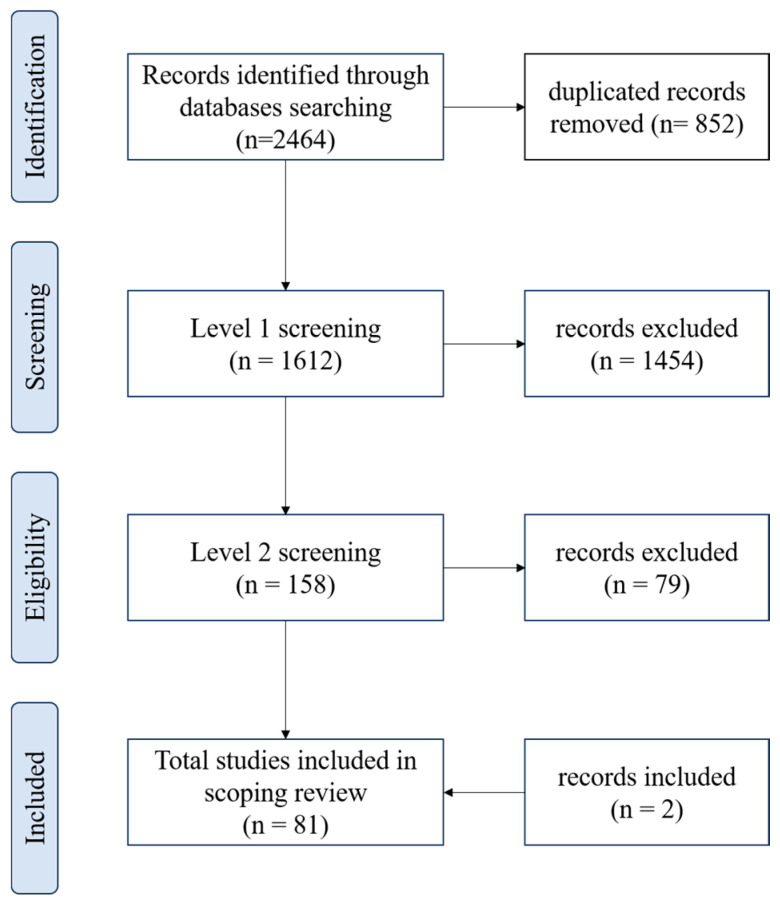
PRISMA (preferred reporting items for systematic reviews and meta-analyses) flowchart showing the selection of studies eligible for a scoping review of the best protocol to treat equine skin wounds.

Figure 1 shows database search results and the flow of articles to inclusion in the scoping review. A total of 2464 papers were found (Scopus: 1355, Web of Science: 959, Pubmed: 150), and 852 were duplicated. After level 1 screening, 158 articles were identified as potentially relevant, and 79 of these records were excluded at level 2 because they did not provide a treatment protocol with immediate clinical applicability. Two studies that did not fully meet the eligibility criteria were added later because the authors considered that they had relevant topics. In the case series published by Lepage et al. [14], some wound types were outside our selection criteria, but 15 wounds were within the interests of this review. In the experimental study published by Wilmink et al. [15], half of the population consisted of ponies (which did not fit the criteria of this review), but as the other half of the population consisted of horses, it was decided to include the study. In total, 81 articles were included in this review.

The greatest number of publications was from the USA (*n* = 21), followed by Brazil (*n* = 18), Australia (*n* = 9), Canada (*n* = 5), Belgium (*n* = 5), France and Italy (*n* = 4), Sweden (*n* = 3), Denmark and Egypt (*n* = 2), Algeria, Austria, Colombia, India, Iraq, Netherlands, New Zealand, and Pakistan (*n* = 1). Most of the articles were in English (*n* = 75), only six were in Portuguese, and none were in French or Spanish. 

**Figure 2:**
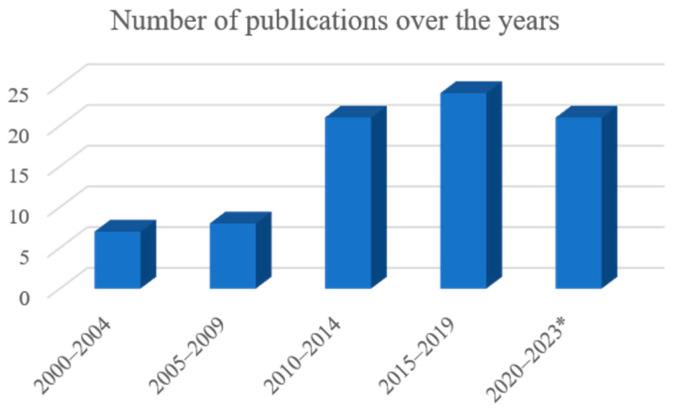
Number of publications per five-year period. * The last period is only 3 years and 9 months.

The 81 articles eligible for the scoping review were published between 2000 and 2023, and the number of articles per year was quite variable, as follows: 2000 (1), 2002 (2), 2003 (3), 2004 (1), 2005 (2), 2006 (1), 2008 (1), 2009 (4), 2010 (2), 2011 (6), 2012 (4), 2013 (7), 2014 (2), 2015 (4), 2016 (5), 2017 (10), 2018 (4), 2019 (1), 2020 (10), 2021 (4), 2022 (3), and 2023 (4). Figure 2 shows the number of publications added over five-year periods. It is possible to observe that the number of articles published on this topic in the early 2000s was much lower than that observed from the second decade onwards. The last group covers a shorter period (3 years and 9 months) because the research was carried out in September 2023.

**Figure 3:**
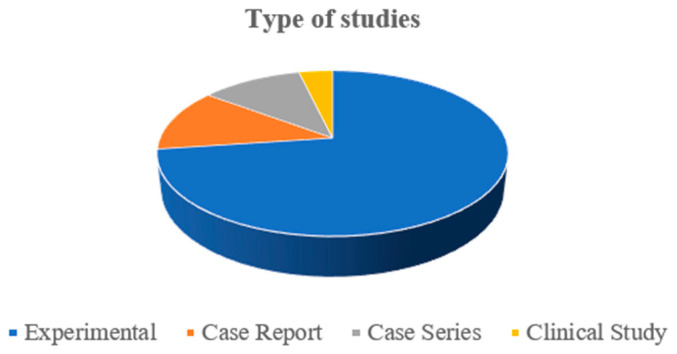
Type of studies included in the scoping review.

The most common type of study was experimental (59/81), in which skin wounds are surgically created to test a new therapy, and the results obtained are compared with control wounds, treated conventionally, or left untreated. The second and third most frequent types of study were case reports (10/81) and case series (9/81), respectively. Both present results from real wound treatments, but there are no control wounds to compare the effectiveness of the new therapy with a standard treatment. Case reports present the treatment of just one animal, while case series report the treatment of at least two horses. The number of clinical studies (real wounds treatment with control wounds comparison) was very small (3/81). Figure 3 shows the distribution of the type of studies in the included manuscripts.

Regarding the anatomical location of the lesions, in 76% of the experimental research, wounds were induced in distal limbs (45/59) and, in fewer studies, in the lumbar region (7/59), glutes (6/59), thorax (4/59), and neck (3/59). In some of these cases, lesions were induced in more than one location. In the case reports, most injuries were also in the distal limbs (60%), and a few were in other regions (back, chest, and proximal front/hindlimb). In 44% of the case series, wounds were in distal limbs. Some articles reported wounds in several locations, and one article did not specify the location of the injuries. In all three clinical studies, wounds were on the distal limb. 

The number of horses evaluated in each study varied greatly among the articles reviewed. Case reports, by definition of our methodology, are articles that report only one animal. The number of participants in the case series varied between 2 and 42. The clinical studies had 18, 29, and 481 horses. In experimental research, the number of horses studied ranged from 1 to 33, the number of wounds ranged from 8 to 100, and the number of wounds per horse (wounds/horse) ranged from 1 to 14. 

The “outcomes measured” extracted from each study showed which tools were used to evaluate the effectiveness of the treatment. Macroscopic assessment of wound healing was the main tool used in all types of publications, and it included wound area measurements, healing rate, granulation tissue, exudate, crust, bleeding, edema, and epithelialization. It is worth mentioning that in some studies, the macroscopic evaluation was objective, with the adoption of well-defined scores for each parameter, blind judgment, and evaluation of areas using software, while in other studies the evaluation was more subjective. In general, case reports, case series, and clinical studies performed gross macroscopic evaluations, and in 50% of case reports it was not clear which parameters were evaluated. In the 59 experimental studies, 52 performed macroscopic evaluation of the healing process, 43 performed histopathological analysis, and 9 studies conducted immunohistochemistry for different markers. Microbiological evaluations, such as bacterial quantification or identification of microorganisms, were reported in seven studies. 

Regarding the outcomes (results obtained with the treatments used), all of the case reports, case series, and clinical studies reported positive results with the treatment used. On the other hand, only 36% of the experimental studies found significantly better results in treated groups compared to control groups, in 51% of the studies the results were similar between the groups, and in 13% the results obtained in control groups were better than in the treated groups. The following tables summarize data extracted from experimental studies (Table 1), case series (Table 2), clinical studies (Table 3), and case reports (Table 4).

## 4. Discussion

Exuberant granulation tissue (EGT) occurs often during second intention wound healing in horses, and the standard treatment of EGT remains focused on single or repeated resection of any tissue that grows above the wound margins [95]. Bader and Eesa [17] concluded that surgical removal of hypergranulation tissue promotes faster healing when compared with wounds undergoing no surgical resection. Caustic materials (copper sulfate ointment 10%, silver nitrate ointment 2%, and red mercury ointment 11%) also suppressed hypergranulation tissue and promoted healing to varying degrees. Silver nitrate ointment caused necrosis, scaling, pain, and lameness. Red mercury ointment showed better results when compared to other caustic materials [17]. A case series published by Lepage et al. [14] showed that maggot debridement therapy can also be recommended in equids for debridement and enhanced healing and its potent antibacterial action, being aware that maggot debridement therapy is not indicated on wounds invaded with a tumor and if bone sequestration is suspected.

The use of bandages has been associated with EGT development. In their study, Berry II and Sullins [18] noted that all wounds treated with bandages exhibited exuberant granulation tissue, whereas none of the un-bandaged wounds showed signs of EGT development. Dart et al. [29] also observed that open wounds began to contract earlier and went through a rapid and more intense period of contraction than bandaged wounds, which produced excessive granulation tissue that required regular resections. Bandaged limb wounds, in addition to developing EGT and showing delayed healing, also had significantly higher biofilm grades compared to un-bandaged limb wounds [45]. According to Anantama et al. [95], the frequency of bandage change may also affect EGT formation. More frequent bandage changes, and therefore more frequent removal of excess exudate, may be beneficial in preventing EGT. Silicone dressings, compared to conventional non-adherent permeable dressings, showed better results as an inhibitor of EGT [32].

Numerous studies have reported positive outcomes with the use of honey in equine wound healing, and all of the included experimental studies utilized Manuka honey [19,20,21,22,52,70]. Two articles demonstrated that the positive effects of Manuka honey occurred in the early stages of the healing process. In 2011, Bischofberger et al. [19] observed that treated wounds remained significantly smaller than control wounds until day 42; however, there was no difference in overall healing time between treatment and control wounds. In another study, it was observed that wounds treated with manuka honey for 12 days or throughout healing were smaller than control wounds until day 35, and there was no difference between the groups in terms of treatment duration [20].

Tsang et al. [70] compared Unique Manuka Factor (UMF) 20 Manuka honey, UMF5 Manuka honey, generic multifloral honey, and a saline control. They observed that wounds treated with UMF20 healed faster than wounds treated with generic multifloral honey and control wounds. However, in 2013, Khiati et al. [90] reported the use of natural Algerian honey in the treatment of a skin wound on a horse’s chest and observed relief of edema and inflammation around the wound, decreased wound exudation, and a reduction in the size of the wound after 2 weeks of treatment. Although the use of generic honey is quite common in equine medicine, for reasons of lower cost and easy acquisition, there are still not enough controlled studies to evaluate its effectiveness. 

Cell therapy was a topic covered in many articles, mainly in experimental studies. Platelet-rich plasma (PRP) and leukocyte-poor platelet-rich plasma (LP-PRP) were the most studied treatments, but oral mucosal mesenchymal stromal cells (OM-MSC), adipose stem cells (ASC), endothelial colony forming cells (ECFCs), epithelial-like stem cells (EpSCs), and peripheral blood stem cells (PBSCs) were also used in some studies. Autologous and allogeneic cells were tested. The most common administration method was injection into the wound margins, followed by topical application in gel, using the culture medium with the vehicle, and encapsulated in polyethylene glycol fibrinogen microspheres. The results found were controversial. Monteiro et al. [54] observed that topical application of autologous PRP did not accelerate or improve the quality of wound healing in horse limbs; contrarily, PRP favored the excessive development of granulation tissue and significantly delayed wound healing. However, Pereira et al. [57] found that the use of PRP in different forms (autologous × homologous, injected × gel) improved the healing process of wounds located in the distal limbs of horses, with autologous PRP gel being the best form of application. 

A single local administration of LP-PRP 12 h after surgical induction of a skin lesion in the gluteal region of horses resulted in significant immunohistochemical expression of collagens, mainly COL III. This greater expression did not result in faster closure of the surgical wound, although microscopically the tissue treated with LP-PRP presented better tissue quality [64,65]. Sajjad et al. [61], in histological analyses, also found increased re-epithelialization and better organization of collagen in wounds treated with PRP. On the other hand, Freitas et al. [36] observed no differences in the histopathological analyses and healing time comparing wounds treated with platelet-rich plasma (PRP), adipose stem cell-conditioned medium (ASC-CM), ASC-CM + PRP, and saline (control).

According to Broecks et al. [23], wounds treated with autologous epithelial-like stem cells (EpSCs) closed significantly faster than control untreated wounds. MSC-treated wounds showed significant differences in wound area, gene expression, and histologic scores, particularly when applied by direct injection into the wound margin [67], and the gel produced by the combination of oral mucosa mesenchymal stromal cells (OM-MSC) or its secretome with hyaluronic acid (HA) showed a positive impact when applied during the early stages of wound healing [31].

Several experimental studies have evaluated the effects of antimicrobials in equine skin wound treatment and, in general, no signs of healing improvement have been reported. Harmon et al. [38] found no differences in the time to wound closure and histological characteristics of wound healing comparing the following treatments: 1% silver sulfadiazine cream, triple antimicrobial ointment (neomycin sulfate, bacitracin zinc, and polymyxin B sulfate), hyperosmolar nanoemulsion at 0.063% [wt:wt] thymol concentration, and control. Berry II and Sullins [18] also found no difference in healing parameters, mean days to healing, percentage of wound contraction, or rate of epithelialization between wounds treated with 1% silver sulfadiazine cream, 1% silver sulfadiazine slow-release matrix, povidone-iodine ointment, and untreated controls; and Bischofberger et al. [21] found delayed healing in wounds treated with BNP commercial antibiotic ointment (bacitracin–neomycin–polymixin B). 

Intravenous regional limb perfusion (IVRLP) treatment for 3 consecutive days using amikacin sulfate did not affect wound size, wound contraction, or healing rate in equine distal limbs [34]. In 2012, Hart et al. [39] tested the use of a subcutaneously placed cross-linked dextran gel impregnated with amikacin, vancomycin, amikacin and clindamycin, and saline (control). The authors concluded that cross-linked dextran gel was a safe, effective alternative local antimicrobial delivery method; however, there were no significant differences in histomorphological scores between treatment and control wounds. 

Treatment with non-steroidal anti-inflammatory phenylbutazone was associated with a significant delay in second intention wound healing. Hussni et al. [43] compared the thoracic and lumbar wounds of horses treated or not with phenylbutazone (4.4 mg/kg IV) and observed through macroscopic and histopathological analysis the inhibitory effect of phenylbutazone on the healing process.

Some studies have investigated the use of biological dressings in equine wounds. These treatments yielded mixed results, making clear recommendations difficult. Gomez et al. [37] compared the use of different biological dressings (split-thickness allogeneic skin dressing, allogeneic peritoneum dressing, and xenogenic porcine small intestinal submucosa dressing) with non-biological dressings, and no significant differences were detected. The biological dressings had no effect on infection, inflammatory response, or healing time, and vascularization was not identified in any of the biological dressings. The treatment of wounds with equine amniotic allografts did not affect the time to healing or histologic quality of the healing, but was associated with increased granulation tissue in distal limb wounds of horses [33,35]. On the other hand, treatment with equine amniotic membrane promoted faster recovery, greater neovascularization, better quality fibroplasia, and less sensitivity to pain than the control group in another study [60]. A case report described the successful use of Nile tilapia (*Oreochromis niloticus*) fish skin applied to the lesion, and it was favored complete re-epithelization of the wound, as well as being considered safe, effective, and low cost, [85].

Hyaluronic acid (HA) has also been investigated as a skin wound healing promoter in horses with controversial results. A commercially available esterified HA fleece under a nonadherent dressing did not show better results than a control dressing [73], but wounds treated with cross-linked HA-based biomaterial (CMHA) gel were significantly smaller on day 31 and healed with higher quality than control wounds [26].

Gallium may be an attractive and novel means of improving equine distal limb wound healing. It is a semi-metallic element that has been shown to possess antimicrobial properties and aid in wound healing in various preclinical models. The use of 0.5% gallium maltolate (GaM) as a topical wound treatment resulted in a more rapid reduction in wound size, reduced formation of exuberant granulation tissue, reduced *S. aureus* bioburden, and improved wound morphology histologically in equine wounds [48].

Recently, a novel hydrogel system has been developed, which incorporates an integrin-binding prosurvival peptide derived from angiopoietin-1, QHREDGS (glutamine-histidine-arginine-glutamic acid-aspartic acid-glycine-serine), into a chitosan–collagen biocomposite material. This “Q-peptide hydrogel” (QH) demonstrated accelerated healing in a diabetic mouse model by promoting rapid re-epithelialization and improved physiological features [96], and in 2021, it was tested by Sparks et al. [66] in horses’ distal limbs wounds, resulting in a higher rate of wound closure, and it was also able to modulate the biomechanical function toward a more compliant healed tissue without observable negative effects. According to the researchers, the use of Q-peptide hydrogel provides a safe and effective means of improving the rate and quality of wound healing.

Phytotherapy was used in some experimental research and also in case/series reports. Reports showed positive results with the use of Ricinus Assept^®^ [93], *Aerva javanica* [75], and *Hypericum perfoliatum* [76]. Experimental research with different types of treatments has had varied results. While Souza et al. [63] confirmed that *Triticum vulgare* cream reduced wound healing time in lumbar and limb wounds, Ribeiro et al. [59] found a significant delay in the final phase of healing in wounds treated with Fitofix^®^. No differences in healing time were observed between control wounds and wounds treated with *Copaifera langsdorffii* [46,50], *Carapa guianensis Aublet* [16], and 1% cannabidiol in unique manuka factor (UMF) 5 manuka honey [52].

The literature regarding some physical treatments has shown improvements in the healing process, mainly avoiding EGT. According to Jann et al. [44], wounds treated with low-level laser therapy (LLLT) healed faster than control wounds, and a significant clinical observation was the absence of exuberant granulation tissue in the laser-treated wounds. Bader and Eesa [17] also found the best results with laser therapy in healing wounds when compared with other methods used (bandage, copper sulfate ointment 10%, silver nitrate ointment 2%, and red mercury ointment 11%). Treatment with extracorporeal shock wave therapy (ESWT) did not accelerate the healing of equine distal limb wounds, but treated wounds had less exuberant granulation tissue and appeared healthier than controls [62]. Link et al. [49] observed evidence of downregulation of TGF-β1, which may decrease the production of granulation tissue in shock wave-treated wounds.

A well-established physical therapy in human medicine that has been described in equine management wounds is negative pressure wound therapy (NPWT). Using our search criteria for this review, we found two case reports [87,89] and two case series [78,80] that used NPWT with positive results. However, Haspeslagh et al. [40] found no long-term advantage in the use of NPWT in noncontaminated or contaminated equine distal limb wound healing. In this experimental study with 40 wounds, no differences between treated and control wounds were observed in terms of wound size, histological parameters, bacterial load, or growth factor concentration. Still in the physiotherapy field, it was investigated whether treatment with pulsating visible red light (λ ≈ 637 nm) and near-infrared (NIR) light (λ ≈ 956 nm) would affect equine wound healing, and the results did not indicate any clinically relevant positive effect compared with no treatment [53]. 

Gas therapy results have been controversial. Ozone was used to treat equine skin wounds through ozonized isotonic sodium chloride solution, ozonized andiroba oil [16], and ozonated sunflower seed oil [30]. Araujo et al. [16] observed no significant differences between treatments and the control group, but Di Filippo et al. [30] concluded that topical application of ozonated sunflower seed oil accelerates acute cutaneous wound repair in horses, preventing hyper-granulation tissue and infection. Topical oxygen therapy (TOT) had no significant effect on wound area, epithelialization, wound contraction, histological or culture results compared with control wounds, and there were no negative effects noted during its use [69]. Hyperbaric oxygen therapy (HBOT) after full-thickness skin grafts applied to wounds promoted less granulation tissue, edema, and neovascularization, but more inflammation. The superficial portion of the graft was less viable than the control, and the authors concluded that this therapy was not indicated.

Some other treatments that have been tested in experimental studies but have not demonstrated improvements in the healing process include Solcoseryl^®^ [15], Solugel^®^ [28], Vulketan^®^ [58], chitosan film [51], activated protein C [21], maltodextrin [42], and interleukin-10 from the Orf virus [72]. Although maltodextrin gel did not accelerate wound healing in the aforementioned research [42], the total percentage of wound healing was significantly higher in maltodextrin–ascorbic acid gel (Multidex^®^) treated wounds compared to the control group in a clinical study [83]. Ketanserin gel (Vulketan^®^) had already been compared with control treatments in a large clinical study in 2004 [82] which reported success in 88% of treated cases; however, the experimental study carried out in 2009 did not confirm the same result.

## 5. Conclusions

Although the literature about equine wound management is very vast, there is still a lack of evidence for the adoption of a treatment protocol considered ideal. The BEVA guidelines for evidence-based medicine on equine wound healing pointed out the overall poor evidence in wound healing studies [8]. There are many treatments with controversial results. Furthermore, the methodology used, and the parameters evaluated, are often different between experimental studies, making comparison difficult. 

Case reports and case series do not have a control group, but could serve to disseminate the results of treatments that are being carried out in equine practice, and compare them with the experimental studies’ results. However, the lack of analyzed parameters compromises the quality of the information disclosed in these types of articles. Many experimental studies present results obtained at specific moments and do not provide information throughout the wound-healing period. This may be a limitation of these studies because some treatments may bring benefits at different stages of the healing process. Furthermore, the total wound healing time is perhaps the most interesting information when choosing a therapeutic protocol, since the main drawback in equine wound management is the prolonged treatment time.

## Figures and Tables

**Table 1:** Experimental studies included in the review, listed in alphabetical order of author’s name.

Authors	Treatment Groups	*n*	Outcomes (Intervention Effects)
Araujo et al., 2017 [16]	Isotonic sodium chloride solution (NaCl 0.9%) (GC)/Ozonized isotonic sodium chloride solution (GO)/Pure andiroba (*Carapa guianensis Aublet*) oil (GAP)/Ozonized andiroba oil (GAO)	5 (40 wounds)	Histopathologic evaluation revealed that wounds from the GAO and GAP had advanced epithelialization. Wound healing times were similar between all groups.
Bader and Eesa, 2011 [17]	Bandage/copper sulfate ointment 10%/silver nitrate ointment 2%/red mercury ointment 11%/laser therapy. Surgical resection of the hypergranulation tissue group and no surgical group for each treatment	20 (40 wounds)	Surgical removal of hypergranulation tissue promoted healing. All caustic material led to depressed hypergranulation tissue and promotion of healing at different degrees. Silver nitrate ointment caused necrosis, slough, pain, and lameness. Red mercury ointment gave the best results when compared with other caustic materials. The laser therapy obtained the best results when compared with other methods used in this study.
Berry II and Sullins, 2003 [18]	1% silver sulfadiazine cream with bandage/1% silver sulfadiazine slow-release matrix with bandage/1% silver sulfadiazine slow-release matrix without bandage/povidone-iodine ointment with bandage/untreated control with bandage/untreated control without bandage	6 (36 wounds)	No difference in healing parameters, mean days to healing, percentage of wound contraction, or rate of epithelialization. All bandaged wounds produced exuberant granulation tissue. None of the unbandaged wounds produced exuberant granulation tissue.
Bischofberger et al., 2011 [19]	Manuka honey/no treatment	8 (16 wounds)	Treatment with manuka honey decreased wound retraction, and treated wounds remained significantly smaller than control wounds until day 42; however, there was no difference in overall healing time between treatment and control wounds.
Bischofberger et al., 2013 [20]	UMF 20 manuka honey applied daily for 12 days/66% manuka honey gel applied for 12 days/gel applied for 12 days/manuka honey gel applied throughout healing/untreated control	10 (100 wounds)	Wounds treated with manuka honey and manuka honey gel for 12 days and throughout healing were smaller than gel control and untreated control wounds until day 35.
Bischofberger et al., 2015 [21]	Activated protein C (APC)/untreated (control)/66% manuka honey gel/commercial antibiotic ointment (bacitracin–neomycin–polymixin B ointment; BNP)/petrolatum (the base for BNP)	6 (66 wounds)	There was no effect of APC on wound size, rate of healing, or overall time to heal. However, compared with control wounds, histological scoring demonstrated enhanced epithelialization (day 4) and angiogenesis (day 11). Wound healing variables for wounds treated with APC, manuka honey gel, and control wounds were not different, and the variables for wounds treated with BNP and petrolatum demonstrated delayed healing.
Bischofberger et al., 2016 [22]	Contaminated wounds treated with manuka honey gel/noncontaminated control wounds/contaminated control wounds	10 (11 wounds)	Manuka honey gel had no significant effect on TGF-β1 and TGF-β3 concentrations or wound bacterial counts, but decreased wound inflammation (days 7, 10), increased angiogenesis (days 2, 7, 10), increased fibrosis and collagen organization (day 7), and increased epithelial hyperplasia (days 7, 10).
Broecks et al., 2015 [23]	Autologous epithelial-like stem cells (EpSCs)/allogeneic EpSCs/Dulbecco’s modified Eagle’s medium (DMEM) as a vehicle treatment/untreated control	5 (60 wounds)	Wounds treated with autologous EpSCs closed significantly faster. Other critical wound-healing parameters, such as granulation tissue, vascularization, and cellular immune response, were significantly improved by both EpSC treatments.
Carter et al., 2003 [24]	PRP gel under regular gauze/saline solution (0.9% NaCl) soaked gauze/dry regular gauze	1 (14 wounds)	PRP gel-treated wounds on day 7 expressed more intense cytokeratin 10 staining. By day 79, the staining was equal in both groups. However, PRP gel-treated wounds at day 79 contained abundant, dense collagen bundles oriented parallel to each other and the overlying epithelium, whereas control tissues contained fewer collagen fibers that were oriented randomly.
Caruso et al., 2022 [25]	Allogenic bone marrow-derived MSCs injected subcutaneously at four equidistant sites on each wound/isotonic saline solution injected (control)	4 (32 wounds)	No difference between the control and treated groups throughout the study.
Dahlgren et al., 2016 [26]	Single application of cross-linked hyaluronic acid-based biomaterial (CMHA) gel/multiple applications of CMHA gel/multiple applications of CMHA film/control (no CMHA)	8 (96 wounds)	Wounds treated with CMHA films decreased to half their original size significantly faster, were significantly smaller on day 31, and healed with higher quality than control wounds.
Dart et al., 2002 [27]	Intramuscular recombinant equine growth hormone/equivalent volumes of sterile water	9 (18 wounds)	Wounds retracted more during treatment and contracted faster after treatment stopped when compared with wounds from untreated horses.
Dart et al., 2002 [28]	Solugel^®^ (25% propylene glycol hydrogel) under dry regular gauze/saline solution (0.9% NaCl) soaked gauze (control)	8 (16 wounds)	Treatment did not affect the total rate of healing, rate of healing during the retraction phase of healing, rate of healing after the retraction phase was complete, or the amount the wounds retracted.
Dart et al., 2009 [29]	Bandaged with a non-occlusive dressing covered by gauze-coated cotton wool that was compressed with adhesive tape/unbandaged	33 (33 wounds)	Bandaged wounds showed greater and more prolonged retraction time and an excess of granulation tissue that required regular trimming. Open wounds began contracting earlier and underwent a rapid and intense period of contraction. There was no difference between groups in the total days to healing or the overall rate of healing.
Di Filippo et al., 2020 [30]	Ozonated sunflower seed oil/pure sunflower seed oil/0.9% sodium chloride (control)	8 (32 wounds)	The ozone group had a significantly smaller wound size and a residual wound area than the control and the oil groups on days 14 and 21. The control wounds and oil wounds had suppurative exudate and the presence of *Streptococcus zooepidemicus*. Exuberant granulation tissue was observed only in the control group on days 14 and 21. Re-epithelialization was observed on day 14 in the ozone group.
Di Francesco et al., 2021 [31]	Hyaluronic acid (HA) gel containing allogeneic equine oral mucosa mesenchymal stromal cells (OM-MSC)/HA gel containing OM-MSC secretome/HA gel alone/no medication	8 (64 wounds)	All wounds healed without adverse effects at day 62. OM-MSC and its secretome had a positive impact on thorax wound contraction. OM-MSC had a positive impact on the contraction and epithelialization of forelimb wounds. No significant difference between wound sites before and after treatment was noted at histological examination.
Ducharme-Desjarlais et al., 2005 [32]	Silicone dressing/conventional nonadherent permeable dressing (control)	5 (20 wounds)	The silicone dressing outperformed the conventional dressing in preventing excessive granulation tissue, enhancing tissue quality. Microvessel occlusion increased with silicone, reducing mutant p53 expression linked to apoptosis inhibition, despite unconfirmed quantitative apoptosis changes via TUNEL.
Duddy et al., 2023 [33]	Wound margins were injected with equine-origin liquid amnion allograft (ELAA)/0.9% NaCl (control)	8 (16 wounds)	No difference was found between the treatment and control groups in either wound area over time or time for wounds to reduce in size by 95%. Exuberant granulation tissue required resection twice (one control wound and one treatment wound).
Edwards-Milewski et al., 2016 [34]	Intravenous regional limb perfusion (IVRLP) using amikacin sulfate/no IVRLP	7 (14 wounds)	No differences were observed between groups for wound size, wound contraction, and healing rate. Mononuclear cell infiltration was greater in the IVRLP group compared with controls.
Fowler et al., 2019 [35]	Equine amniotic allografts (eAM)/eAM control/occlusive silicone gel dressings/nonadherent dressings	8 (32 wounds)	Treatment of wounds with eAM did not affect the time to healing or histologic quality of the healing compared with other groups, but was associated with increased granulation tissue production early in the study, particularly on day 7.
Freitas et al., 2023 [36]	Platelet-rich plasma (PRP)/adipose stem cell-conditioned medium (ASC-CM)/ASC-CM + PRP/saline solution (control)	8 (32 wounds)	Comparing all treatments, no differences were observed in the histopathological analyses. Healing time was similar among all treatments.
Gomez et al., 2003 [37]	Split-thickness allogeneic skin dressing (STS)/allogeneic peritoneum dressing (P)/xenogenic porcine small intestinal submucosa dressing (PSIS)/nonbiologic dressing (NASP)	5 (60 wounds)	No significant difference was detected. Biological dressings had no effect on infection, inflammatory response, or healing time. Vascularization was not identified in any of the biological dressings.
Harmon et al., 2017 [38]	1% silver sulfadiazine cream (SSC)/triple antimicrobial ointment (TAO [neomycin sulfate, bacitracin zinc, and polymyxin B sulfate])/hyperosmolar nanoemulsion (HNE) at 0.063% [wt:wt] thymol concentration/no topical medication applied (control)	8 (32 wounds)	Time to wound closure and histologic characteristics of wound healing did not differ among groups.
Hart et al., 2012 [39]	Amikacin-/vancomycin-/amikacin and clindamycin-impregnated gel placed subcutaneously/saline (control)	11 (11 wounds)	There were no significant differences in histomorphological scores between treatment and control incisions.
Haspeslagh et al., 2020 [40]	NPWT/calcium alginate dressings	10 (40 wounds)	In noncontaminated wounds, wound size was not significantly different between NPWT and control wounds at later healing stages. In contaminated wounds, no differences between treatments were observed in wound size, histological parameters, bacterial load, or growth factor concentration.
Holder et al., 2008 [41]	Hyperbaric oxygen therapy (HBOT) + full-thickness skin grafts applied to fresh wounds/no HBOT	6 (48 wounds)	Histologic examination of biopsy specimens revealed that grafts treated with HBOT developed less granulation tissue, edema, and neovascularization, but more inflammation. The superficial portion of the graft was also less viable than the superficial portion of those not treated with HBOT.
Howard et al., 2018 [42]	Maltodextrin gel/no treatment	8 (16 wounds)	Maltodextrin did not accelerate wound healing. Based on observations from this study, maltodextrin should be discontinued once granulation tissue has filled the wound bed to prevent hypergranulation tissue formation.
Hussni et al., 2010 [43]	Phenylbutazone (4.4 mg/kg) IV + local Dakin’s solution/distilled water (2.2 mL/100 kg) IV + local Dakin’s solution (control group)	10 (40 wounds)	The time to complete healing was significantly greater in the phenylbutazone group than in the control group. Thoracic and lumbar wound contraction was decreased in the phenylbutazone group. Gross and histopathology analysis showed the inhibitory effect of phenylbutazone on second intention wound healing when compared to the control group.
Jann et al., 2012 [44]	Low-level laser therapy LLLT/no treatment (control)	8 (8 wounds)	Wounds treated with LLLT healed faster than the control wounds. Wounds treated with LLLT were completely epithelialized at day 80 after surgery. Control wounds were not epithelialized at postoperative day 80. A significant clinical observation was the absence of exuberant granulation tissue in the laser-treated wounds.
Jørgensen et al., 2017 [45]	Bandaged limb wounds/un-bandaged limb wounds/un-bandaged shoulder wounds	9 (81 wounds)	Bandaged limb wounds developed EGT and displayed delayed healing, while the shoulder and unbandaged limb wounds healed normally. Significantly higher biofilm grades were identified in bandaged limb wounds compared to un-bandaged limb wounds.
Kauer et al., 2020 [46]	Base cream (Lanette) vehicle (BC)/base cream containing hydroalcoholic extract from 10% *Copaifera langsdorffii* (copaiba) leaves (HE)/base cream containing oil-resin extracted from 10% copaiba bark (OR)/saline solution 0.9% control (SS)	6 (48 wounds)	Topical treatments of oil-resin and hydroalcoholic extract formulations of copaiba did not reduce healing time within the first 7 days after surgical wound induction. After 14 and 21 days, the copaiba-based treatments, especially the oil-resin treatment, helped to improve the clinical aspects of the lesions.
Kelleher et al., 2013 [47]	Silver sodium zirconium phosphate polyurethane semi-occlusive foam (SPF) dressing/absorbent dressing (control)	5 (10 wounds)	SPF-treatment wounds had significantly decreased wound area and decreased granulation tissue scores when evaluated at <30 days and over the 60 day study, although complete wound healing times were not significantly different. Bacteria were cultured from all wounds at varying times throughout the study.
Lawless et al., 2020 [48]	Gallium maltolate (GaM) 0.5% in a petroleum base (Aquaphor Beiersdorf Inc)/drug-vehicle petroleum ointment (control)	6 (48 wounds)	The use of 0.5% GaM as a topical wound treatment resulted in a more rapid reduction in wound size, reduced formation of exuberant granulation tissue, reduced *S. aureus bioburden*, and improved wound morphology histologically in equine wounds.
Link et al., 2013 [49]	Extracorporeal shock wave therapy (ESWT)/untreated (control)	14 (60 wounds)	ESWT had no significant effect on the expression of the evaluated growth factors and histologic examination of the intact skin. There was evidence of downregulation of TGF-β1 in treated wounds. No significant effect on expression of FGF-7, IGF-1, PDGF, and VEGF was found in shock wave-treated wounds.
Lucas et al., 2017 [50]	10% copaiba oil/0.9% sodium chloride (control	8 (32 wounds)	No significant differences were observed between the groups.
Martins et al., 2013 [51]	Chitosan film/sodium chloride 0.9% (control)	4 (16 wounds)	The chitosan film did not interfere with healing time but promoted granulation tissue.
McIver et al., 2020 [52]	1% cannabidiol in unique manuka factor (UMF) 5 manuka honey/UMF 5 manuka honey/UMF 20 manuka honey/saline	6 (30 wounds)	There was no difference in wound area, daily healing rate, or days to complete healing between treatment groups.
Michanek et al., 2020 [53]	Pulsating visible red light (λ ≈ 637 nm) and near-infrared (NIR) light (λ ≈ 956 nm)/untreated control	8 (16 wounds)	The wound area and degree of swelling did not differ between the treatment and control groups on any day. There was a significant difference (*p* = 0.03) in healing time between control (49.0, 95% CI = 35.4–62.6 days) and treated wounds (51.8, 95% CI = 38.7–64.8 days).
Monteiro et al., 2009 [54]	Platelet-rich plasma (PRP) and bandaged/bandage only (control)	6 (36 wounds)	PRP favored excessive development of granulation tissue and significantly slowed wound healing. Transforming growth factor-β1 had a 1.6-fold higher concentration in treated wounds. Histologic, biomechanical, and gene expression data did not differ significantly between groups.
Mund et al., 2021 [55]	Allogeneic cord blood-derived MSCs suspended in 50% HypoThermosol FRS intravenously/50% HypoThermosol FRS alone	12 (72 wounds)	Three of the six treatment horses and one of the six control horses experienced minor transient reactions. Treatment did not accelerate wound closure or improve histologic healing. Treatment decreased wound size and decreased all measured cytokines except TGF-beta 3.
Oliveira Jr. 2011 [56]	Sunflower seed oil/saline solution (control)	6 (48 wounds)	Treated wounds had higher contraction than the control group and took less time to heal. Wounds treated with sunflower seed oil presented with a more characteristic arrangement of collagen fibers and fibroblasts, reduced inflammatory cells, and a formed capillary bed.
Pereira et al., 2017 [57]	Injection subcutaneously under the wound edges of 10 mL of autologous PRP (PRPaut)/10 mL of homologous PRP (PRPhom)/10 mL autologous PRP in gel form covering skin defects/10 mL of saline solution (control group)	8 (32 wounds)	PRP in different forms was beneficial in improving the healing process of wounds located in the distal limbs of the horses, and autologous PRP gel was the best form.
Ribeiro et al., 2009 [58]	Ketanserin gel (Vulketan^®^)/no treatment	8 (16 wounds)	No difference between control wounds and ketanserin-treated wounds over the course of 56 days.
Ribeiro et al., 2013 [59]	Commercial herbal medicine spray (Fitofix)/no treatment	8 (16 wounds)	The topical application of this herbal combination and propolis did not affect cicatrization in the first 4 weeks. After 10 weeks, wound healing was faster in the control group.
Rosa et al., 2022 [60]	Equine amniotic membrane (EAM)/washing with water and neutral detergent (control)	6 (12 wounds)	Treatment with EAM promoted faster recovery, greater neovascularization, better quality fibroplasia, and less sensitivity to pain than the control group.
Sajjad et al., 2023 [61]	Autologous PRP gel/sterile saline and povidone-iodine solution (ASD, antiseptic dressing)	20 (20 wounds)	In the PRP wounds, there was a highly significant increase in re-epithelization and the collagen was well organized. Malondialdehyde (MDA) concentration was decreased in the PRP wounds. No difference in catalase (CAT) activity.
Silveira et al., 2010 [62]	Extracorporeal shock wave therapy (ESWT) and bandage/bandage only (control)	6 (60 wounds)	Control wounds appeared more inflamed and had higher scores for exuberant granulation tissue. Treatment did not affect wound size or area of neo-epithelialization. No difference was found for any of the histologic or immunohistochemical variables between groups.
Souza et al., 2006 [63]	*Triticum vulgare* cream from the first day/*Triticum vulgare* cream from day 5/no cream (control)	6 (24 wounds)	*Triticum vulgare* cream intensified neovascularization, repaired cell migration, and stimulated fibroblast multiplication and collagen production, reducing the wound healing time.
Souza et al., 2015 [64]	Leukocyte-poor platelet-rich plasma (LP-PRP) administered at each edge of the wounds/no treatment	7 (14 wounds)	The administration of a single dose of LP-PRP 12 h after induction of the wound in horses did not influence the formation of collagens I and III.
Souza et al., 2017 [65]	Leukocyte-poor platelet-rich plasma (LP-PRP) injected into wound margins/no treatment injected	8 (16 wounds)	No difference was observed between the time required for wound closure in the two groups. General microscopic evaluation revealed that the majority of the treated wounds showed better healing variables in sections analyzed after complete macroscopic closure of the wound.
Sparks et al., 2021 [66]	Q-peptide hydrogel (QH)/Q-peptide hydrogel repeated application (RQH)/peptide-free hydrogel (H)/no treatment (E)	10 (80 wounds)	A single treatment with Q-peptide hydrogel resulted in a higher rate of wound closure and was able to modulate biomechanical function toward a more compliant healed tissue without observable negative effects.
Texton et al., 2017 [67]	Equine mesenchymal stem cell (MSC) injected into wound margins/saline injection/MSCs embedded in an autologous fibrin gel applied topically to the wound bed/blank fibrin gel	6 (36 wounds)	Allogeneic MSC therapy was shown to facilitate wound healing. MSC-treated wounds showed significant differences in wound area, gene expression, and histologic scores.
Tóth et al., 2011 [68]	LHP^®^ (1% hydrogen peroxide) cream/petrolatum/untreated	10 (30 wounds)	LHP^®^-treated wounds healed faster than petrolatum and controls. No difference was observed in healing time between petrolatum and controls. LHP^®^ showed lower scores for bacteria and neutrophils compared to petrolatum. *Staphylococcus aureus* and *Streptococcus zooepidemicus* were found only in petrolatum-treated and untreated wounds.
Tracey et al., 2014 [69]	Topical oxygen therapy (TOT)/no treatment	4 (16 wounds)	TOT treatment did not affect wound area, epithelialization, wound contraction, or histological or culture results. There were no negative effects noted during its use.
Tsang et al., 2017 [70]	Unique Manuka Factor (UMF) 20 manuka honey/UMF5 manuka honey/generic multifloral honey (GH)/saline control	8 (32 wounds)	There were differences in mean days to complete healing. Wounds treated with UMF20 healed faster than wounds treated with GH and the control wounds.
Wilmink et al., 2000 [15]	Solcoseryl^®^ (a protein-free, standardized dialysate/ultrafiltrate derived from calf blood)/no treatment	10 (40 wounds)	Solcoseryl^®^ stimulated healing in the first 4 weeks by provoking a greater initial inflammatory response, faster contraction, and faster formation of granulation tissue. Subsequently, it inhibited healing because it significantly delayed epithelialization and caused protracted inflammation.
Winter et al., 2020 [71]	Endothelial colony forming cells (ECFCs) alone/ECFCs encapsulated in poly(ethylene) glycol fibrinogen microspheres (PEG-Fb MS)/MS alone/serum only	6 (48 wounds)	Wounds that were treated with ECFCs with or without PEG-Fb encapsulation had increased vascularization acutely and decreased neutrophilic and macrophagic inflammation chronically. There were no effects of ECFC or ECFC/MS treatment on other measured parameters.
Wise et al., 2018 [72]	Subcutaneous injection of saline with orf virus interleukin-10 (ovIL-10) and vascular endothelial growth factor-E (VEGF-E)/subcutaneous injection of saline	4 (36 wounds)	Viral protein treatment did not accelerate healing at either location or limit EGT formation in limb wounds. Treatment of limb wounds did, however, increase epithelialization and angiogenesis without dampening inflammatory cell infiltration or gene expression.
Witte et al., 2009 [73]	Commercially available esterified HA fleece under a nonadherent dressing/nonadherent dressing alone (control)	6 (72 wounds)	There was no difference in mean percentages of total wound healing, epithelialization, and wound contraction between the control and treatment groups.

**Table 2:** Case series included in the review, listed in alphabetical order of author’s name.

Authors	Treatment	*n*	Outcomes (Intervention Effects)
Chevalier and Pearson, 2023 [74]	Amorphous silicate dressing	11	None of the wounds required granulation bed debridement. There were no complications associated with the treatment. All referring veterinarians and owners were satisfied with the healing.
Dedar et al., 2020 [75]	Leaf extract of *Aerva javanica* topical spray	15	All cases (15) of exuberant growth of granulation tissue in horses treated with leaf extract of *Aerva javanica* showed suppression of EGT growth.
Giudice et al., 2017 [76]	Oil prepared from the aerial parts of St. John’s Wort (*Hypericum perfoliatum*)	6	Topical application of the oil of St. John’s Wort determined a significant improvement in skin lesions in all of the horses involved in the study.
Janet D. Varhus, 2013 [77]	Bioelectric device	10	Enhanced re-epithelialization, decreased inflammation, reduced pain and scar formation, hair regrowth, and favorable cosmetic outcomes were observed in the 10 presented cases.
Launois et al., 2021 [78]	Negative Pressure Wound Therapy (NPWT)	42	In 69% of the cases, healing was considered satisfactory at discharge. The procedure was well tolerated, except in one horse who showed signs of discomfort at the first application.
Lepage et al., 2011 [14]	Maggots applied directly or a closed polyester net with absorbent hydrophilic polyurethane foam (LarveE BioFOAMT)	41	In 38 cases, a favorable outcome was reached in less than one week. In five cases, a second maggot application was necessary to reach the desired level of wound healing. Some discomfort was recorded in seven horses between 24 and 72 h of treatment.
Olofsson et al., 2016 [79]	Honeybeespecific lactic acid bacteria (LAB) formulation	10	Rapid and painless healing of chronic equine wounds was observed with honeybee LAB formulation treatment.
Rijkenhuizen et al., 2005 [80]	Vacuum-assisted wound closure (VAC) in combination with micrografting method	2	After 3 weeks, graft acceptance was at least 75%, wound contraction had decreased the size of the wound (20%), epithelialization was present, and the loose flap had become firmly attached. After 5 weeks, the wounds were epithelialized and showed functional and cosmetically acceptable healing.
Spaas et al., 2012 [81]	Intradermal injections of peripheral blood stem cells (PBSCs) at the wound edges, and an intravenous injection into the jugular vein	4	In all cases, tissue overgrowth was visible within 4 weeks after PBSC injection, followed by the formation of crusts and small scars in the center of the wound, with hair regeneration at the edges.

**Table 3:** Clinical studies included in the review, listed in alphabetical order of author’s name.

Authors	Treatment Groups	*n*	Outcomes (Intervention Effects)
Engelen et al., 2004 [82]	Ketanserin gel (Vulketan^®^/ethacridin lactate (Rivanol)/cream containing malic, benzoic, and salicylic acids (MBS)	481	The ketanserin group was successful in 88% of cases. Wounds treated with ketanserin were two and five times more likely to heal successfully than those treated with MBS or ethacridin lactate, respectively.
Helal et al., 2022 [83]	Maltodextrin-ascorbic acid gel (Multidex^®^)/1% povidone-iodine solution before being dressed with Multidex^®^/1% povidone-iodine solution	18	The total wound healing percentage was increased between the study groups. The use of maltodextrin/ascorbic acid gel resulted in considerable wound contraction, rapid epithelialization, and complication-free wound healing.
Wilmink et al., 2020 [84]	Topical probiotic treatment (probiotic suspension containing strains of *Lactobacillus acidophilus, Bifidobacterium animalis subsp lactis, Lactobacillus paracasei subsp paracasei*)/saline	29	The mean relative wound area of the probiotic-treated wounds decreased faster than saline-treated wounds and did not cause a systemic inflammatory response.

**Table 4:** Case reports included in the review, listed in alphabetical order of author’s name.

Authors	Treatments	*n*	Outcomes (Intervention Effects)
Costa et al., 2020 [85]	Nile tilapia (*Oreochromis niloticus*) fish skin applied to the lesion	1	The skin of tilapia was of low cost and was considered safe and effective, favoring complete re-epithelization of this wound.
El Dine et al., 2016 [86]	Silver nanoparticles (AgNPs) in combination with visible blue light	1	Signs of improvement after 1 week when the tissues partially healed and the amount of exudates decreased. The condition further improved after 2 weeks of continuous treatment, and the wound was free from infection. The wound completely healed after 4 weeks.
Florczyk and Rosser, 2017 [87]	Negative Pressure Wound Therapy (NPWT)	1	Vacuum-assisted closure facilitated the management of a large degloving infected, severely exudating wound with tissue loss in the dorsal aspect of the tarsus.
Iacopetti et al., 2012 [88]	Autologous platelet-rich gel (PRG)	1	The wound healed rapidly and completely within 5 months of the first PRG treatment, without chronic effects or formation of exuberant tissue granulation, and with minimal scarring.
Jordana et al., 2011 [89]	Vacuum-assisted closure (VAC) therapy in combination with skin punch grafting	1	VAC therapy resulted in nearly perfect punch graft survival and significant enhancement in the healing of a large distal limb wound.
Khiati et al., 2013 [90]	Algerian honey	1	Relief of edema and inflammation around the wound, decreased wound exudation, disappearance of infection, and observable decrease in wound surface after one week, and a significant reduction in the size of the wound after two weeks of treatment.
López and Carmona, 2014 [91]	Autologous PRP and platelet-poor plasma (PPP) injected into the foci of the surrounding areas of the lesion	1	No complications were observed with the PRP treatment. The case described reported fast granulation, wound contraction, and epithelialization.
Martins et al., 2018 [92]	Small partial-thickness skin grafts	1	The treatment was shown to provide good recovery and healing of extensive wounds in horses, provided that due care is taken in the postoperative period in relation to dressings and antimicrobial medication.
Peres et al., 2015 [93]	Castor oil-based phytotherapy (Ricinus Assept^®^)	1	During the 24th week of treatment, the wound was fully recovered.
Varasano et al., 2018 [94]	4% formaldehyde solution	1	Reductions in bleeding and exuberant granulation tissue were observed.

## Data Availability

The data presented in this study are available on request from the corresponding author.
